# Experimental Studies on the Thermal Properties and Decomposition Course of a Novel Class of Heterocyclic Anticancer Drug Candidates

**DOI:** 10.3390/ijms24076190

**Published:** 2023-03-24

**Authors:** Marta Worzakowska, Małgorzata Sztanke, Krzysztof Sztanke

**Affiliations:** 1 Department of Polymer Chemistry, Institute of Chemical Sciences, Faculty of Chemistry, Maria Curie-Skłodowska University in Lublin, 33 Gliniana Street, 20-614 Lublin, Poland; 2 Department of Medical Chemistry, Medical University of Lublin, 4A Chodźki Street, 20-093 Lublin, Poland; 3 Laboratory of Bioorganic Compounds Synthesis and Analysis, Medical University of Lublin, 4A Chodźki Street, 20-093 Lublin, Poland

**Keywords:** TG/DTG/DSC, TG/FTIR/QMS, thermal stability, thermal decomposition course, radical mechanism, annelated asymmetrical triazines, heterocyclic anticancer drug candidates

## Abstract

The experimental studies on the thermal properties and decomposition course of a novel class of potential anticancer drugs (**1**–**5**) containing in their heterobicyclic structures the asymmetrical triazine template were performed with the use of differential scanning calorimetry (DSC) and simultaneous thermogravimetry/differential scanning calorimetry (TG/DTG/DSC) coupled online with Fourier transform infrared spectroscopy (FTIR) and quadrupole mass spectrometry (QMS) in inert and oxidizing conditions. All the compounds were thermally characterized in detail for the first time in this article. The DSC studies proved that the melting points of the tested compounds depended on the position and type of the substituent at the phenyl moiety, whereas they did not depend on the furnace atmosphere. All the tested polynitrogenated heterocycles proved to be molecules with high thermal stability in both atmospheres, and most of them (**1**, **3**–**5**) were more stable in oxidizing conditions, which indicated the formation of a more thermally stable form of the compounds when interacting with oxygen. The simultaneous TG/FTIR/QMS analyses confirmed that their pyrolysis process occurred in one main stage resulting in the emission of volatiles such as NH_3_, HNCO, HCN, CO, CO_2_, H_2_O, NO_2_, aromatic amine derivatives, alkenes (for compounds **1**–**5**), and HCl (for the compound **5**). On the other hand, the oxidative decomposition process was more complicated and proceeded in two main stages leading to the emission of NH_3_, CO_2_, CO, HCN, HNCO, H_2_O, some aromatics (for compounds **1**–**5**), HCl (for compounds **3**–**5**) as well as the additional volatiles such as N_2_, NO_2_, NH_2_OH, and (CN)_2_. The type of the formed volatiles indicated that the decomposition process of the studied heterocycles under the influence of heating was initiated by the radical mechanism. Their decomposition was related to the symmetric cleavage of C–N and C–C bonds (inert conditions) and additional reaction of the volatiles and residues with oxygen (oxidizing conditions).

## 1. Introduction

**Figure 1:**
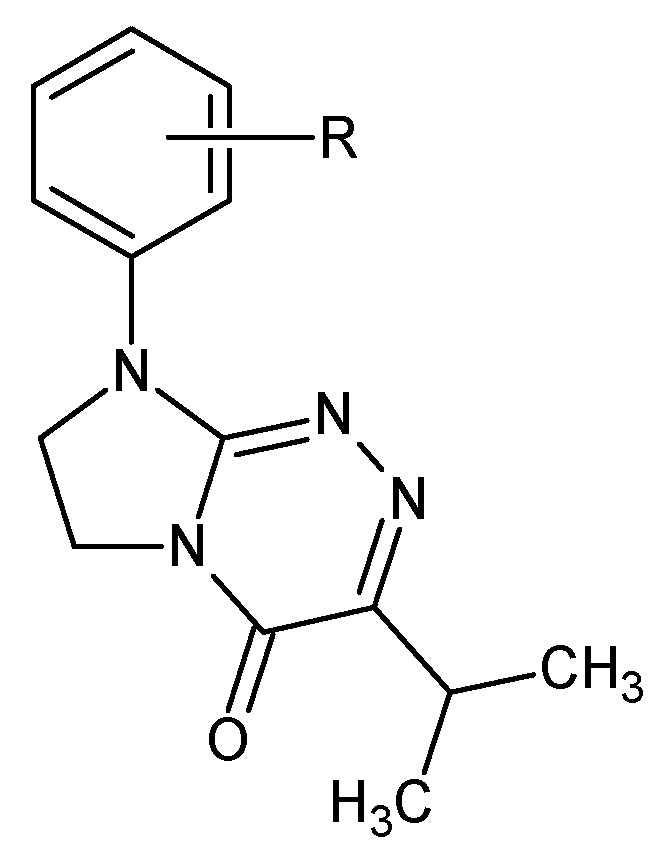
Structures of the investigated heterocycles: **1**. R = H; **2**. R = 4-CH_3_; **3**. R = 3-Cl; **4**. R = 4-Cl; **5**. R = 3,4-Cl_2_. The particular derivatives differ in the substitution pattern (denoted as R) at the phenyl ring.

The anticancer active annelated asymmetrical triazines (**1**–**5**, Figure 1), that have been synthesized in our laboratory, belong to antimetabolite-type molecules that may be irreversible inactivators of specific enzymes [1]. The molecular structures of these innovative heterocycles have previously been established, whereas their experimental lipophilicities (derived from the retention behavior in reversed-phase high-performance liquid chromatography on different columns, including those imitating biosystems), structural and electronic descriptors have been correlated strongly with important pharmacokinetic descriptors, resulting in highly predictive QSAR relationships suggesting their high bioavailability [1,2]. A three-dimensional (3D) structure of compound **2** has recently been established by X-ray crystallography. The crystal data for this molecule—which have been deposited in the Cambridge Crystallographic Data Centre as a supplementary material CCDC 2131634—are available for research purposes [3]. Because of the confirmed in vitro antiproliferative effects against human tumors of the breast, cervix, and lung, the title compounds may have the potential usefulness in the treatment of these tumors according to their disclosed medical application [4]. Noteworthy is that most of the molecules caused stronger antitumor effects than pemetrexed, i.e., the clinically used anticancer agent [1,4]. It was proven that their antiproliferative action was related to the activation of apoptotic caspases, playing a key role in both the initiation and execution phases of programmed cell death [5]. It is worth emphasizing that the *para*-chloro-substituted compound (**4**) reveals the least toxicity to non-cancerous cells [1], the molecules **1**–**4** prove to be safe for zebrafish (*Danio rerio*), and all the heterocycles (**1**–**5**) are completely non-toxic for red blood cells [5], which makes them promising drug candidates in the preclinical phase of drug development. Furthermore, in an ex vivo model of oxidatively stressed erythrocytes, the majority of molecules under investigation showed protective effects, which were better or comparable to those of the commonly used antioxidants, such as ascorbic acid and trolox [1,4].

A number of bioorganic compounds containing a core structural component of asymmetrical triazine, i.e., 1,2,4-triazine, found utility as molecular pharmaceutics [6,7]. Nevertheless, a literature search revealed that there were only a few scientific reports on the thermal analysis of medicines structurally based on the 1,2,4-triazine system [8,9] as well as those containing the template of 2-methyl-3-sulfanyl-1,2-dihydro-1,2,4-triazine-5,6-dione linked via a methylene bridge with a cephem-based hybrid structure [10] or the 1,2,4-triazin-4(3*H*)-one nucleus incorporated into the structure of diazolo-annelated triazinone [11]. Moura Ramos and Diogo used differential scanning calorimetry and thermally stimulated depolarization currents to study the thermal transitions in the solid state of an antiepileptic drug prone to amorphization, lamotrigine [8]. In turn, Fasihi et al., used the thermodynamic Hildebrand equation to calculate the crystal-liquid fugacity ratio and developed a new pharmacokinetic-thermodynamic model based on the experimentally derived thermodynamic properties of lamotrigine to predict its intestinal absorption [9]. Chadha et al., used the solution calorimetry as the thermal technique for quantification of an amorphous content/crystallinity of ceftriaxone sodium (the β-lactam cephalosporin antibiotic possessing a broad spectrum of antimicrobial action) and reported a suitable correlation between the enthalpy of dissolution of ceftriaxone and its amorphous content [10]. However, Attia et al., published studies on the purity, thermal stability, and chemical degradation mode of a sulphonamide containing the imidazo[5,1-*f*][1,2,4]triazin-4(3*H*)-one nucleus, vardenafil hydrochloride (a selective inhibitor of cGMP-specific phosphodiesterase type 5 used as a therapeutic agent in erectile dysfunctions), and performed semi-empirical molecular orbital calculations that were helpful in identifying the weakest bonds broken during its thermal degradation [11].

Furthermore, there are several scientific articles concerning the thermal analysis of pharmacologically or energetically important 1,2,4-triazines as well as 1,2,4-triazines condensed with five-membered azole systems [12,13,14,15,16,17,18,19,20,21,22]. Masłowska and Jaroszyńska studied the stages of thermal decomposition of the pharmacologically active diethyl 2,2′-(3-thioxo-2,3-dihydro-1,2,4-triazine-5,6-diyl)diacetate [12]. Sikorska-Iwan and Modzelewska-Banachiewicz determined the thermal stability range of antimicrobially active methyl (3,4-disubstituted-5-oxo-4,5-dihydro-1,2,4-triazin-6(1*H*)ylidene)acetates and identified the volatile decomposition products emitted during their pyrolysis [13]. Dong et al., characterized the thermal properties of 1,2,4-triazine nitrogen-rich salts bearing a geminal dinitromethyl group and reported their energetic properties superior to that of 2,4,6-trinitrotoluene (TNT), an explosive material [14]. Schulze et al., disclosed the thermal stability and sensitivity data for an energetic material candidate, i.e., 4-amino-3,7,8-trinitropyrazolo[5,1-*c*][1,2,4]triazine [15]. Han et al., investigated the thermal decomposition process of tetrazolo[1,5-*b*][1,2,4]triazine and provided a justification for its applicability as an energetic material [16]. In turn, Zhao et al., studied the thermal stability and decomposition pathways of structurally diverse derivatives of tetrazolo[1,5-*b*][1,2,4]triazine and proved that the vast majority of them reveal a higher detonation efficiency than TNT [17]. Bartyzel et al., studied the thermal properties and mechanism of thermal decomposition of fused (1,2,4-triazin-3-yl)carbohydrazides with significant antitumor activities [18]. Worzakowska et al., investigated the thermal stability range and thermal properties of anticancer active condensed 1,2,4-triazinones possessing the nitrophenyl or trifluoromethyl moiety and explained their decomposition mechanism by means of combined thermal techniques [19,20]. Łyszczek et al. [21] and Bartyzel et al. [22] studied the thermal properties and thermal stability ranges for pharmacologically active condensed 1,2,4-triazinones bearing an additional five-membered heteroaromatic substituent, such as thienyl or furanyl.

This prompted us to extend our thermal investigations conducted on a number of pharmacologically relevant molecules to another structurally different class of patented and published heterocycles, potential anticancer agents (**1**–**5**) [1,2,3,4,5]. The privileged template of 3-amino-4*H*-5-oxo-1,2,4-triazine (present in the tautomeric structure of 6-azaisocytosine) is incorporated in their molecules. The investigated compounds are of particular interest as both thermal properties and thermal degradation pathways of all these molecules with the prospective utility in medicine remain unidentified to date. Thermal stability is an important property that should be assessed in the preclinical phase of drug development. The high thermostability allows for avoiding the problems associated with the storage and processing of newly developed medicines.

The present study analyzes the thermal behavior of a new class of heterocyclic molecules by the use of simultaneous thermogravimetry/differential scanning calorimetry (TG/DTG/DSC) coupled with Fourier transform infrared spectroscopy (FTIR) and quadrupole mass spectrometry (QMS). These thermal analysis techniques allow investigation of the calorimetric and mass changes of the heated sample and are recommended as suitable for a detailed thermal characterization of pharmaceutics as well as drug candidates in the preclinical phase of drug development. The main goal of the present investigation is to determine the range of thermal stability of new compounds, as well as their thermal decomposition course (combined with the qualitative assessment of the gases released during heating) and decomposition enthalpies in inert and oxidizing conditions. The subsequent purpose is to evaluate the influence of the structure of heterocyclic molecules on their melting points and melting enthalpies using the DSC and to document the solid–liquid phase transition of each compound described by the DSC peak in order to check and confirm its purity. The novelty of this investigation is the assessment of the thermal stability range and thermal properties of a novel class of anticancer drug candidates, the identification of the volatile products emitted during their decomposition, and the elucidation of the major thermal decomposition pathways under inert and oxidizing conditions. The presented studies are of great importance in a detailed thermal characterization of these potential anticancer agents because they enable the evaluation of their purity, thermal properties, and events (characterized by an enthalpy change and temperature range), decomposition course, and mechanism. Such studies constitute a significant contribution to the current state of knowledge on the decomposition pathways of thermally stable heterocyclic drug-like molecules, especially those of small molecular mass. The research results are important from a practical point of view and, therefore, would be helpful in determining the optimal conditions for the storage and processing by the pharmaceutical industry of these potential drugs and in assessing their impact on the environment during thermal utilization. The results of this thermal analysis will be useful if these compounds are approved as pharmaceuticals.

## 2. Results and Discussion

### 2.1. Melting Temperatures of Compounds ***1***–***5*** Evaluated Based on the DSC Method

**Figure 2:**
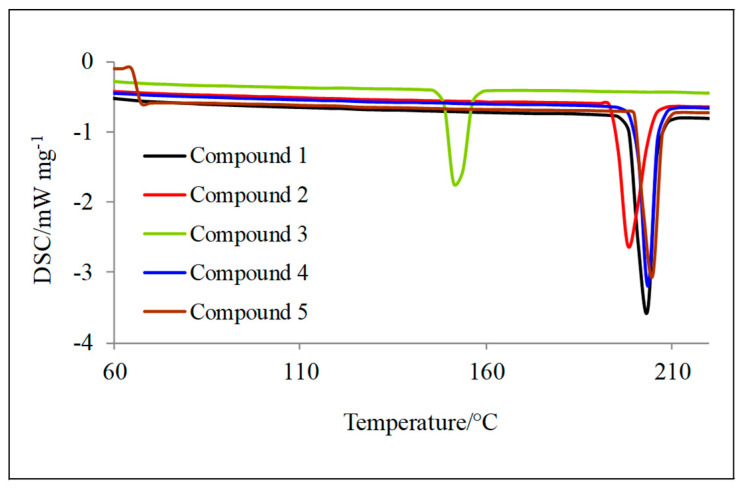
The DSC curves for compounds **1**–**5** (a helium atmosphere).

The melting temperatures—the onset melting temperature (*T*_onset_) and the temperature where the DSC peak maximum is observed (the maximum melting temperature, *T*_peak_) of the tested compounds—were evaluated by use of the DSC method in both atmospheres (a helium and a synthetic air) (Figure 2, Table 1).

All of the investigated compounds (**1**–**5**), which have been recrystallized from methanol/dimethylformamide mixtures, melt within one narrow temperature range, which is manifested by the presence of an endothermic peak on their DSC curves. The solid–liquid phase transition of each compound is described by one sharp peak. It means that all the tested compounds are pure substances, suitable for pharmaceutical applications. The previous spectroscopic, chromatographic, and crystallographic investigations [1,2,3] also confirmed that these heterocyclic molecules are homogeneous and crystalline substances of high purity. Therefore, these crystalline compounds may exhibit higher physical and chemical stability than amorphous substances. The tested molecules melt at different temperatures, the values of which depend on the type of substituent attached at the phenyl moiety, and therefore on their structure. The parent structure, i.e., the compound **1**—containing no substituents at the phenyl moiety—melts at ca. 202 °C in both furnace atmospheres. The introduction of an electron-donating methyl group in the *para* position to the phenyl moiety causes a slight decrease in the melting point. Hence, a drop in the melting temperature by about 5 °C is observed for the compound **2** compared to the melting point of a parent structure. In turn, the presence of one electron-withdrawing chlorine substituent on the phenyl moiety causes a decrease in the melting point (the compound **3**) compared to the melting point of compound **1** or almost the same value (the compound **4**) as observed for the compound **1**. As it is noted among monosubstituted halogenates (**3** and **4**), the lowest melting point is characteristic for the chloro derivative substituted by in the *meta* position (**3**) and the highest one for the chloro derivative substituted in the *para* position (**4**). The highest melting point of all halogen derivatives is observed for the compound **5** (about 204 °C), containing two chlorine substituents at the phenyl moiety, one in the *meta* position and another in the *para* position. In addition, the furnace atmosphere does not affect the melting temperatures of the tested molecules. However, the furnace atmosphere affects the melting enthalpy (Δ*H*) values of the studied compounds (Table 1). The estimated values of Δ*H* in inert conditions are from 46.5 J/g to 81.8 J/g. In turn, the received Δ*H* values in oxidizing conditions are from 122.3 J/g to 154.6 J/g. The highest Δ*H* value for compound **1**—having no substituent at the benzene ring—is observed in both atmospheres (81.8 J/g (an inert atmosphere) and 154.6 J/g (an oxidizing atmosphere)). The replacement of a hydrogen atom at the benzene ring by one or two chlorine substituents leads to a decrease in the melting enthalpy in both used furnace atmospheres. This means that less heat energy is required to convert a unit mass of a solid at its melting point into a liquid without an increase in temperature for the compounds containing at least one chlorine substituent at the phenyl moiety as compared to the heat energy evaluated for both compound **1** without a substituent and compound **2** containing a methyl group in the *para* position.

### 2.2. Thermal Stability of Compounds ***1***–***5*** in Inert Conditions

**Figure 3:**
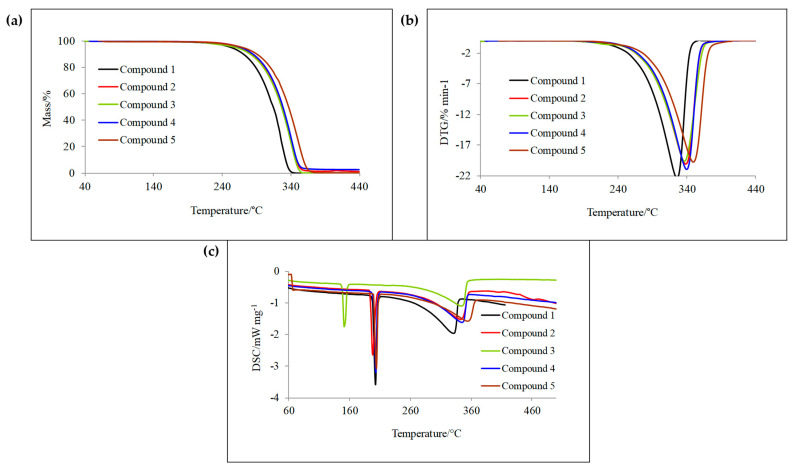
The TG (**a**), DTG (**b**), and DSC (**c**) curves of compounds **1**–**5** (a helium atmosphere).

The TG/DTG curves of the tested compounds collected in inert conditions are presented in Figure 3. In addition, the TG/DTG data such as the initial decomposition temperature (*T*_5%_), the peak maximum temperature (*T*_max1_), the mass loss (Δ*m*_1_), the residual mass at 450 °C (rm), and the decomposition enthalpy (Δ*H_dec_*), are placed in Table 2. All the studied compounds decompose in one stage in an inert atmosphere. Hence, one peak of decomposition is observed on their DTG curves. Their decomposition begins at a temperature above 250 °C. The values of the initial decomposition temperature (*T*_5%_) depend on the type of substituent attached to the phenyl moiety. The *T*_5%_ is found to be 253 °C for parent compound **1**. The *para*-placement of a methyl substituent at the phenyl moiety increases *T*_5%_ to 266 °C. In addition, the compounds with one chlorine substituent in the *meta* or *para* position (**3** and **4**, respectively) show a slight increase in their thermal stability. Among all the monosubstituted halogenates, compound **4**, having a chloro group in the *para* position, is described by the highest thermal stability. Its thermal stability is exactly the same (266 °C) as that of compound **2**, having the *para*-methyl substitution. However, compound **5** with two chlorine substituents, one in the *meta* position and another in the *para* position, is characterized by the highest thermal stability (269 °C) of all the tested molecules in an inert atmosphere.

In addition, the differences in the decomposition enthalpy values (Δ*H_dec_*) are clearly observed. The Δ*H_dec_* values are dependent on the type of substituent at the phenyl moiety (Table 2). Compounds **3** and **4**, containing at least one chlorine substituent at the phenyl moiety, are characterized by lower Δ*H_dec_* values than compounds **1** and **2** without chlorine atoms in their structure. In addition, the lowest Δ*H_dec_* value is observed for compound **5**, containing two chlorine substituents. This indicates that the attendance of the electron-withdrawing substituent/substituents in the structure of the tested compounds reduces the strength of the intermolecular and intramolecular forces, and thus, less heat energy is needed to decompose them.

As mentioned earlier, all the compounds were almost completely decomposed in one stage but over a wide temperature range with a maximum temperature of above 320 °C (*T*_max1_). This means that the cleavage of the bonds present in their structure takes place in a wide range of temperatures, which is caused by the differences in their decomposition energy.

### 2.3. Decomposition Mechanism of Compounds ***1***–***5*** in Inert Conditions

**Figure 4:**
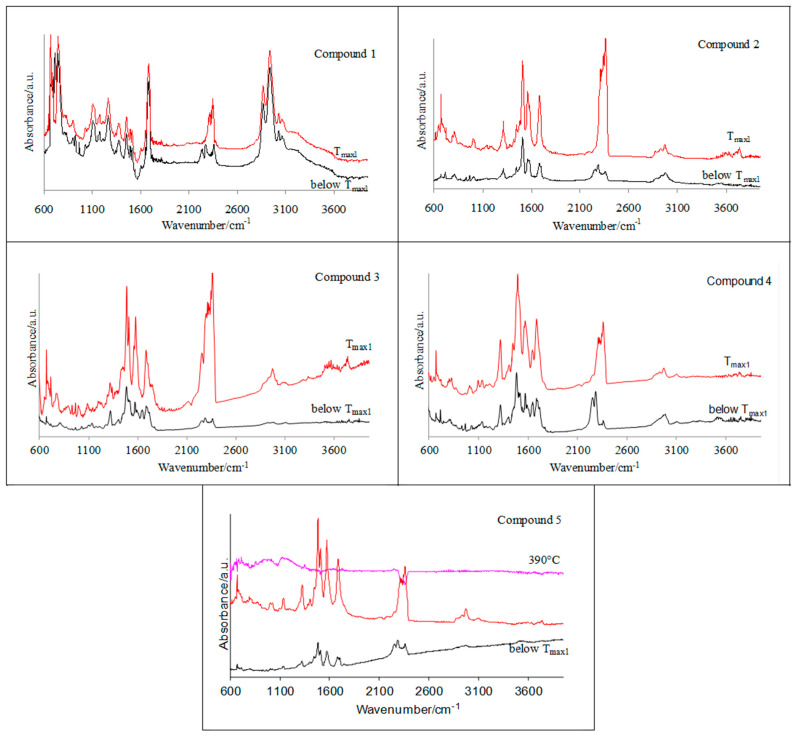
The FTIR spectra of volatiles emitted under the heating of the tested compounds **1**–**5** (a helium atmosphere).

**Figure 5:**
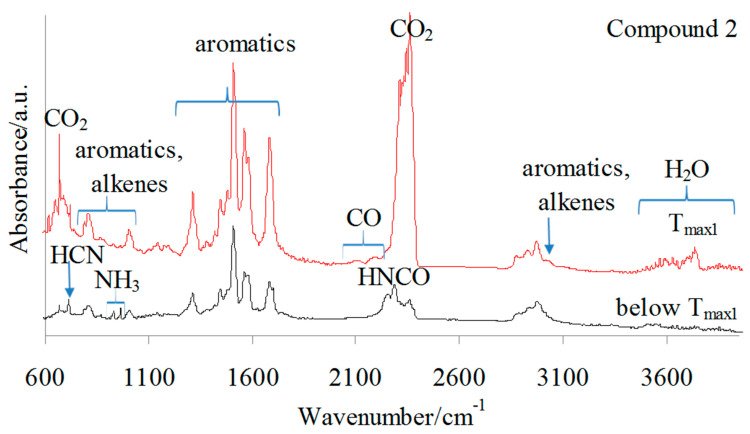
The enlarged FTIR spectrum for compound **2** with marked decomposition products.

The gaseous FTIR spectra collected together with the TG/DTG analyses at the temperature of 20 °C lower than the peak maximum temperature *T*_max1_ (marked on the curves as below *T*_max1_) and the spectra gathered at *T*_max1_ are presented in Figure 4. In addition, the FTIR spectrum with marked decomposition products for the selected compound **2** is shown in Figure 5.

As it is well visible, the emission of the volatiles connected with the decomposition of the central core system is confirmed. Therefore, in the spectra the attendance of a mixture of gaseous decomposition products is observed. The emission of ammonia (NH_3_) among gaseous decomposition products emitted during the heating in inert conditions is clearly visible. The emission of ammonia as one of the decomposition products is confirmed by the presence of two absorption bands, one at 931 cm^−1^ and another at 966 cm^−1^, which are characteristic for the deformation vibrations of the N–H groups [23,24,25,26]. In addition to the emission of NH_3_, the formation of hydrogen cyanide (HCN) (the absorption signal at 713 cm^−1^), cyanic acid (HNCO) and its derivatives (RNCO) (the absorption bands with the maximum centered at 2270 and 2290 cm^−1^) [27,28,29,30], carbon dioxide (CO_2_, peaks at 2300–2365 cm^−1^ (valence vibration) and 669 cm^−1^ (deformation vibration)), carbon monoxide (CO, 2050-2270 cm^−1^), and water (H_2_O, several, jagged, weak signals at the range of 3450–4000 cm^−1^) is also observed (Figure 4 and Figure 5). The emission of NH_3_, HNCO, RNCO, and HCN suggests the random breaking of the C–N bonds in the tested compounds and the formation of intermediate species. However, it should be noted that the emission of HNCO and RNCO starts below *T*_max1_. The emission of CO_2_ and CO is visible later, at *T*_max1_. Along with the formation of NH_3_, HCN, HNCO, and RNCO, the creation of some aromatic compounds and alkenes is confirmed. The emission of aromatics is proven by the presence of the following absorption bands: the stretching vibrations of the C_Ar–H_ at 3025–3090 cm^−1^, the deformation vibrations of the N–H at 1638–1677 cm^−1^, the stretching vibrations of the C_Ar_=C_Ar_ at 1500–1590 cm^−1^, the stretching vibrations of the C–N at 1255–1317 cm^−1^ and the out-of-plane deformation vibrations of the N–H and the C_Ar–H_ at 640–900 cm^−1^ [31]. The position of the absorption bands and the presence of the characteristic vibrations of the N–H bonds indicate the creation of aromatic amine derivatives as one of the decomposition products. In the case of the compound **1** (the parent structure), the emission of aniline is confirmed based on the position of the absorption bands on the FTIR spectra (the stretching vibrations of the C_Ar–H_ at 3010 and 3052 cm^−1^, the deformation vibrations of the N–H at 1646 and 1677 cm^−1^, the stretching vibrations of the C_Ar_=C_Ar_ at 1520 cm^−1^, the stretching vibrations of the C–N at 1260 cm^−1^ and out-of-plane deformation vibrations of the N–H and the C_Ar–H_ at 665–931 cm^−1^).

In turn, in the case of compound **2**, the most likely aromatic gaseous product emitted during its thermal decomposition is *p*-toluidine. For compounds **3**–**5**, containing the chlorine atom/atoms in their structures, the formation of the corresponding chloroanilines is demonstrated by the presence of the absorption bands characteristic for the N–H vibrations within the appropriate wavelength range for *meta*- (compounds **3** and **5**) and *para*- (compounds **4** and **5**) chloroanilines (Figure 4). The emission of some other aromatics with a lower molecular mass, which is difficult to identify from the FTIR spectra due to the similar position of the absorption bands as for the main decomposition products, should also be taken into account [29,32].

In addition, the attendance of the absorption bands at 3035–3040 cm^−1^ characteristic for the stretching vibrations of the =C–H may suggest the emission of alkenes, as presented in Figure 4. The type of volatiles emitted during the heating of the tested compounds suggests that their pyrolysis process is based on a radical mechanism. The random cleavage of the C–N and the C–C bonds, after they have reached enough energy for decomposition, produces radicals that can react with each other in a variety of ways. Therefore, it is important to remember that in the case of radical reactions, decomposition products of higher molecular masses (conjugated products) may be formed, which are low-volatile compounds, and therefore, it is not possible to analyze them using the FTIR method, because they can condense in the transfer line tube.

**Figure 6:**
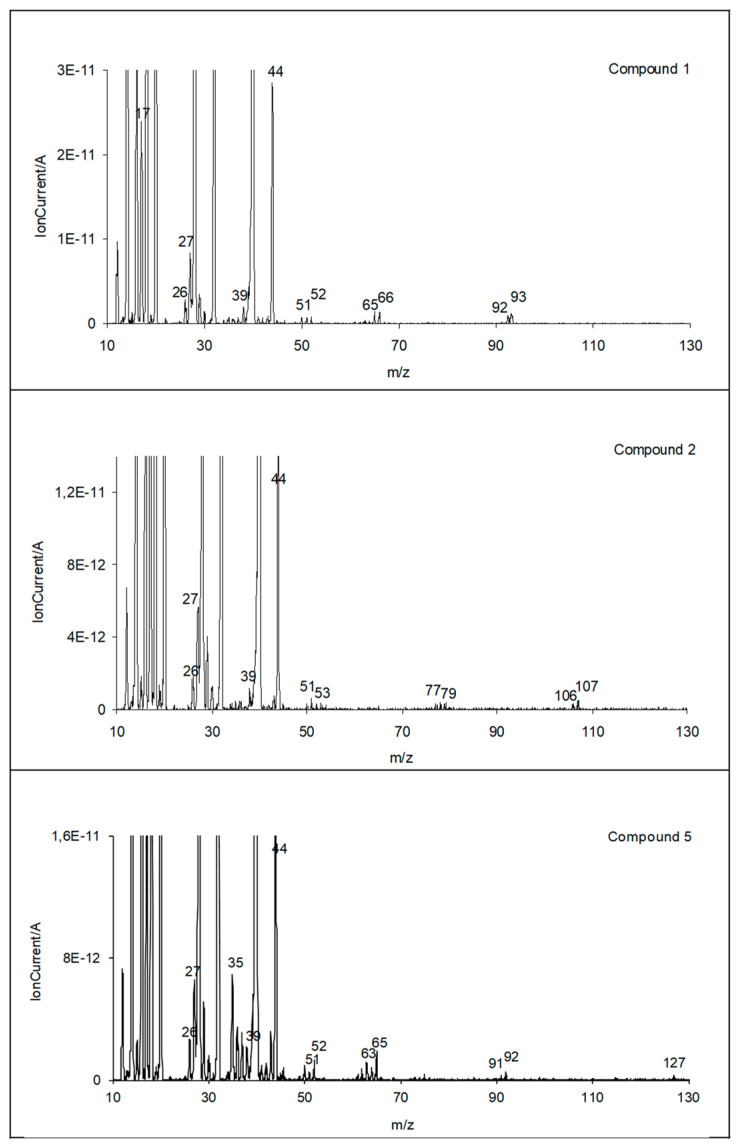
The QMS spectra of volatiles emitted under the heating of the selected compounds (an inert atmosphere).

The emission of the above-mentioned volatiles is also confirmed based on the QMS analysis (Figure 6). According to the MS database, the ionization of NH_3_ leads to the formation of the following species: NH_3_^+^ (*m*/*z* 17), NH_2_^+^ (*m*/*z* 16), NH^+^ (*m*/*z* 15), and N^+^ (*m*/*z* 14). All these *m*/*z* ions are present in the QMS spectra collected at a temperature below *T*_max1_ for all the tested compounds. The ionization of H_2_O gives the following *m*/*z* ions: H_2_O^+^ (*m*/*z* 18), OH^+^ (*m*/*z* 17), and O^+^ (*m*/*z* 16). The *m*/*z* ions 17 and 16 are the characteristic *m*/*z* ions for both NH_3_ and H_2_O. The main difference is the presence of the *m*/*z* ion 18. This *m*/*z* ion is characteristic and proves the emission of water. HCN, during ionization, forms main ions with the following *m*/*z* 27 (HCN^+^), 26 (CN^+^), 12 (C^+^), and 14 (N^+^). The QMS analysis confirms the attendance of *m*/*z* ions characteristic of hydrogen cyanide formed as a result of its ionization. In addition, the release of CO_2_ is proven by the presence of the *m*/*z* ions 44 (CO_2_^+^), 28 (CO^+^), 16 (O^+^), and 12 (C^+^) in the QMS spectrum. The *m*/*z* ion 28 is also characteristic of the emission of CO (CO^+^). The formation of CO is well visible from the gaseous FTIR spectra. Thus, the QMS analysis is an additional confirmation that this inorganic gas is one of the decomposition products.

The attendance of the *m*/*z* ions 43 (HNCO^+^) and 42 (NCO^+^) may indicate the formation of isocyanic acid. However, it is suspected that the emitted HNCO may react with the resulting H_2_O. This leads to the emission of NH_3_ and CO_2_ as the final products [33]. In addition, the formation of methyl or ethyl isocyanate is suspected. However, the lack of the *m*/*z* ions 57 (CH_3_NCO^+^), 56 (CH_2_NCO^+^), 55 (CHNCO^+^), 70 (CH_2_CH_2_NCO^+^), and 71 (CH_3_CH_2_NCO^+^) excludes the formation of isocyanic acid derivatives. 

The creation of aniline occurs as a result of the pyrolysis process of the N–C bonds present in the structure of the parent compound **1**. The aromatic compounds undergo the benzyl fragmentation process. The ionization of aniline (*m*/*z* 93, C_6_H_7_N^+^) leads to the loss of a proton of the amino group, and aniline is converted to C_6_H_6_N^+^ (*m*/*z* 92) and then by the subsequent loss of one HCN molecule to the cyclopentadienyl ion (C_5_H_5_^+^, *m*/*z* 65). Aniline (*m*/*z* 93) may also lose one HCN molecule and form ion C_5_H_6_^+^, *m*/*z* 66. The elimination of one acetylene molecule from the cyclopentadienyl ion causes the formation of the ion C_3_H_3_^+^ (*m*/*z* 39). In addition, the elimination of two acetylene molecules from the benzene ring leads to the creation of the ions C_4_H_3_^+^ (*m*/*z* 51) and C_4_H_4_^+^ (*m*/*z* 52). The presence of these *m*/*z* ions in the QMS spectrum confirms the formation of aniline as a result of the decomposition process of compound **1**.

In the case of compound **2**, the expected gaseous product of its thermal decomposition is *p*-toluidine (C_7_H_9_N^+^, *m*/*z* 107). According to the literature data, alkyl anilines undergo tropylic cleavage, resulting in the formation of amino-tropylium ion (C_7_H_8_N^+^, *m*/*z* 106) [34]. Due to the loss of one HCN molecule, the amino-tropylium ion goes into C_6_H_6_^+^ (*m*/*z* 78), C_6_H_5_^+^ (*m*/*z* 77), and C_6_H_7_^+^ (*m*/*z* 79). However, the loss of two molecules of acetylene by the amino-tropylium ion leads to the creation of C_4_H_3_^+^ (*m*/*z* 51), C_4_H_4_^+^ (*m*/*z* 52), and C_4_H_5_^+^ (*m*/*z* 53). The type of the *m*/*z* ions found in our experimental QMS spectra is in accordance with the literature data and confirms the creation of *p*-toluidine (Figure 6). 

The emission of chloroanilines as the most likely decomposition products of compounds **3**–**5** is also confirmed by the presence of the following *m*/*z* ions: 127 (C_6_H_6_N^35^Cl^+^), 129 (C_6_H_6_N^37^Cl^+^), 65 (C_5_H_5_^+^), 92 (C_6_H_5_NH^+^), 64 (C_5_H_4_^+^), 63 (C_5_H_3_^+^) and 39 (C_3_H_3_^+^) in the QMS spectra.

**Scheme 1:**
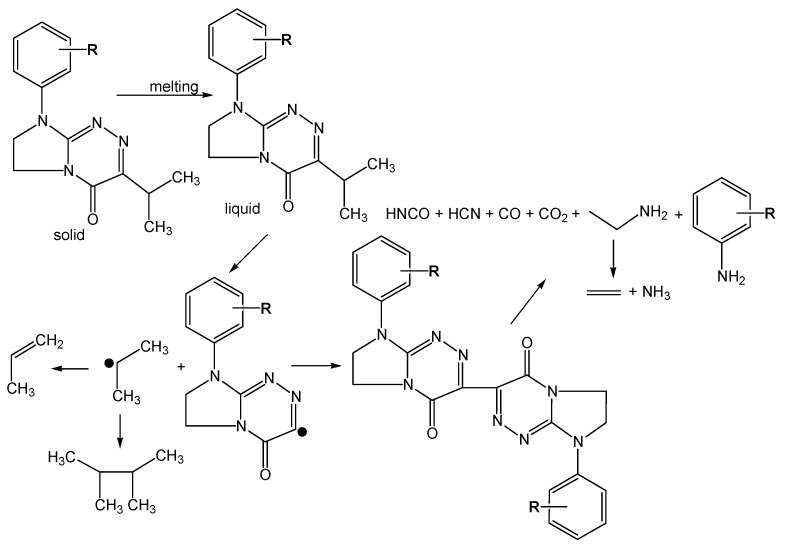
The pyrolysis products of the tested compounds formed in inert conditions.

In addition, the formation of hydrochloric acid (HCl) as a decomposition gaseous product of the compound **5** is proven by the attendance of the *m*/*z* ions 35 (^35^Cl^+^), 36 (H^35^Cl^+^), 37 (^37^Cl^+^), and 38 (H^37^Cl^+^) in the QMS spectrum collected at 390 °C. The main pyrolysis products of the tested compounds formed in inert conditions are presented in Scheme 1.

### 2.4. Thermal Stability of Compounds ***1***–***5*** in Oxidizing Conditions

**Figure 7:**
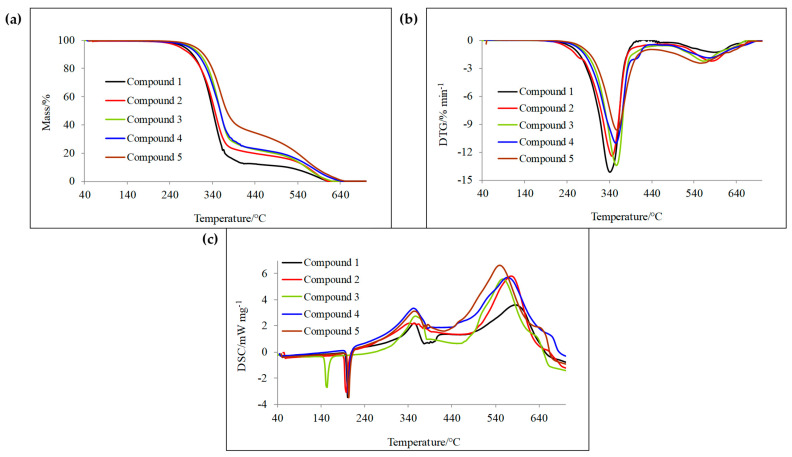
The TG (**a**), DTG (**b**), and DSC (**c**) curves of compounds **1**–**5** (a synthetic air atmosphere).

The TG/DTG curves for all the compounds obtained under oxidizing conditions are shown in Figure 7, and the TG/DTG data are presented in Table 3.

As it is well seen, the thermal stability of the studied compounds, described as the temperature at which a 5% mass loss is observed, is significantly higher for most of the tested molecules (**1**, **3**–**5**) than their thermal stability in an inert atmosphere. Only compound **2,** containing a methyl group in the *para* position of the phenyl moiety, shows a thermal stability lower by 2 °C as compared to its thermal stability in an inert atmosphere. As it is confirmed, the thermal stability for all the compounds is above 260 °C in oxidizing conditions. The compounds with the electron-withdrawing chlorine atom/atoms (**3**–**5**), as well as the unsubstituted structure (**1**), are more thermally stable than molecule **2**, bearing an electron-donating *para*-methyl substitution. 

The parent compound **1** starts to decompose at 275 °C. Its thermal stability in oxidizing conditions is ca. 22 °C higher than its thermal stability in inert conditions. The introduction of one chlorine substituent in the *meta* or *para* position of the phenyl moiety (compounds **3** or **4**, respectively) leads to an increase in their thermal stability as compared to that of compound **1**. Similarly, the presence of two chlorine substituents at the phenyl ring in molecule **5** contributes to a significant increase in its thermal stability (300 °C). The thermal resistance of this compound is 31 °C higher in oxidizing conditions as compared to its thermal stability in inert conditions. In the presence of synthetic air, the initial decomposition of the tested compounds is delayed by their interactions with oxygen. The higher initial decomposition temperatures of the tested compounds indicate that oxygen appears to interact with the condensed phase material to form a more thermally stable product. The stabilizing effect of oxygen may be explained by the reaction of the studied compounds with oxygen to form more thermally stable radical species than initial compounds. This means that oxygen acts as an inhibitor of the decomposition process of the tested compounds, delaying their decomposition process by increasing the decomposition activation energy of newly formed radical species.

The tested compounds proved to be thermally stable materials at temperatures much higher than ambient conditions. This thermal property would be of great importance in the case of their approval as medicines. In light of the current knowledge, it is known that in the case of molecular pharmaceuticals that are stable at a temperature much higher than ambient temperature, their storage in the temperature range from 20 °C to 45 °C has an irrelevant impact on their shelf life [35,36]. The studied molecules demonstrate usefulness as drug candidates due to their favorable thermal properties (i.e., the high thermal stability and the sharp DSC peaks associated with their solid–liquid phase transition process confirming their purity), which would be desirable during their storage and processing by the pharmaceutical industry.

As it is visible on the basis of the conducted studies, the decomposition of all the tested compounds takes place in two main stages in an oxidizing atmosphere. The first decomposition stage is observed to ca. 440 °C with *T*_max1_ from 333 °C to 358 °C and with the main mass loss above 65%. In turn, the second decomposition stage spreads from ca. 440 °C to ca. 650 °C with *T*_max2_ above 555 °C and with the mass loss from 9.8% to 35.1%. 

Interestingly, in the DSC curves collected in an oxidizing atmosphere, the presence of exothermic signals is observed in the temperature ranges where the DTG curves show the two stages of decomposition. In addition, the enthalpy changes in the first decomposition stage (Δ*H_dec_*_1_) and in the second decomposition stage (Δ*H_dec_*_2_) are placed in Table 3. It is clearly seen that the Δ*H_dec_*_1_ values are significantly lower than the Δ*H_dec_*_2_ values. This indicates a different nature of the processes in both decomposition stages. It is expected that mainly chemical reactions between the intermediate decomposition products and/or oxygen take place and lead to the emission of volatiles with a different chemical structure than those observed in an inert atmosphere during the first decomposition stage. During the second decomposition stage, the combustion processes are the main processes.

### 2.5. Decomposition Mechanism of Compounds ***1***–***5*** in Oxidizing Conditions

**Figure 8:**
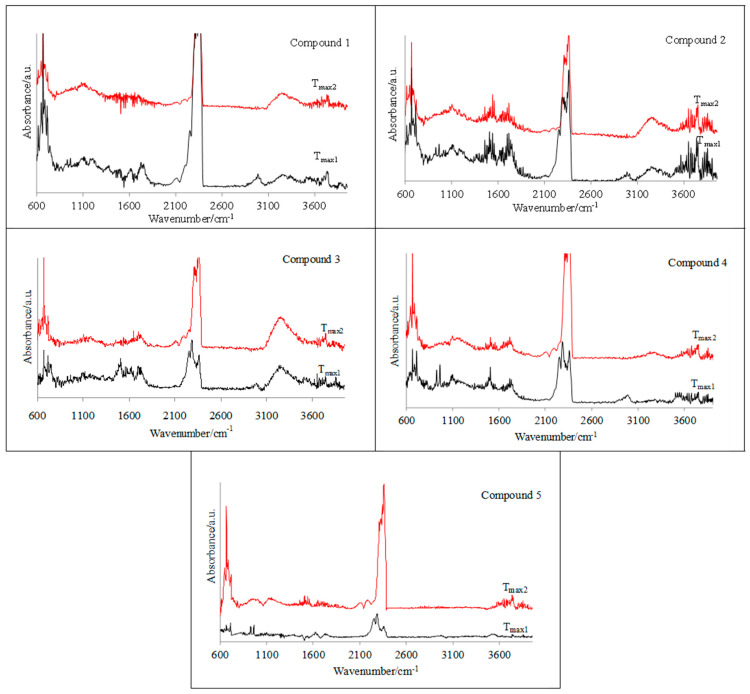
The gaseous FTIR spectra of volatiles emitted under the heating of the tested compounds in an oxidizing atmosphere.

The gaseous FTIR spectra for the tested compounds collected at *T*_max1_ and *T*_max2_ are presented in Figure 8. 

As it is well visible, at *T*_max1_, the emission of NH_3_ (931 cm^−1^ and 966 cm^−1^), CO_2_ (699 cm^−1^ and 2300–2365 cm^−1^), CO (2050–2270 cm^−1^), HCN (713 cm^−1^), HNCO (2270–2290 cm^−1^), and H_2_O (3450–4000 cm^−1^ and 1300–1950 cm^−1^) is clearly confirmed. Moreover, on the gaseous FTIR spectra collected under the first decomposition stage, the absorption signals of a very low-intensity characteristic for aromatic compounds at 640–865 cm^−1^ (the out-of-plane deformation vibrations of the N–H and the C_Ar–H_), 1307–1350 cm^−1^ (the stretching vibrations of the C–N), 1500–1610 cm^−1^ (the stretching vibrations of the C_Ar_=C_Ar_), 1615–1620 cm^−1^ (the deformation vibrations of the N–H), 1700–1733 cm^−1^ (the stretching vibrations of the C=O) and 2850–3030 cm^−1^ (the stretching vibrations of the C–H and the C_Ar–H_) appeared. This indicates the chemical reactions of the formed volatiles, such as aromatic compounds with oxygen. As a result, H_2_O, CO_2_, CO, N_2_, Cl_2_, and HCl (compounds **3**–**5**) can be created. In addition, small amounts of aromatic compounds are emitted.

**Figure 9:**
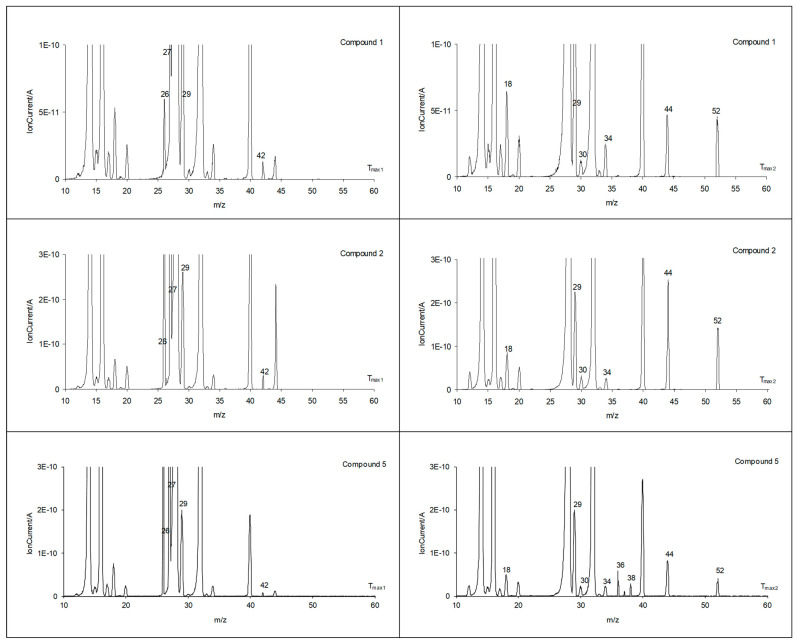
The QMS spectra collected at *T*_max1_ and *T*_max2_ for the selected compounds in an air atmosphere.

These observations were confirmed based on the QMS analysis (Figure 9). 

**Scheme 2:**
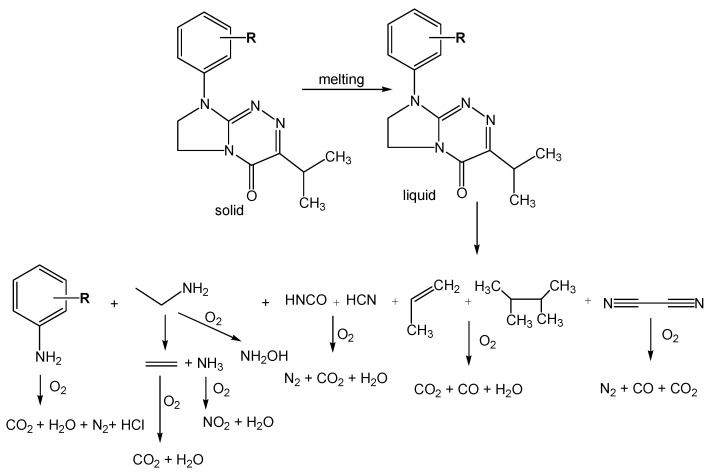
The decomposition mechanism: the products of the tested compounds formed in oxidizing conditions.

At *T*_max1_, the formation of NH_3_ is proven by the presence of the following *m*/*z* ions: 17 (NH_3_^+^), 16 (NH_2_^+^), 15 (NH^+^), and 14 (N^+^). The emission of H_2_O is also visible as the *m*/*z* ions: 18 (H_2_O^+^), 17 (OH^+^), and 16 (O^+^). The QMS analysis indicates also the formation of CO_2_ and CO (*m*/*z* 44–CO_2_^+^, *m*/*z* 28–CO^+^, *m*/*z* 16–O^+^ and *m*/*z* 12–C^+^). The high intensity of the *m*/*z* ions 28 and 14 and the appearance of the *m*/*z* ion 29 may be an indication that volatiles containing nitrogen in their structure burn, emitting nitrogen (N_2_) [37]. The formation of nitrogen dioxide (NO_2_) is possible as a result of the oxidation of some ammonia. The FTIR spectra show the presence of the absorption bands for this nitrogen oxide at 1610–1640 cm^−1^. NO_2_ creation is also confirmed based on the attendance of the following *m*/*z* ions: 30 (NO^+^), 46 (NO_2_^+^), 16 (O^+^), and 14 (N^+^). The occurrence of the *m*/*z* ions 30, 32, 33, and 34 indicates the emission of hydroxylamine as a reaction product between amines and oxygen. The emission of HCN and HNCO is confirmed based on the presence of the *m*/*z* ions: 42, 27, 26, 12, and 14 in the first decomposition stage. According to the literature survey, these compounds can react with oxygen forming CO_2_, CO, H_2_O, and N_2_ [38,39,40]. In turn, in the second decomposition stage, the emission of the same volatiles as in the first decomposition stage (CO, CO_2_, H_2_O, N_2_, NO_2_, and hydroxylamine (NH_2_OH)), as a result of further reactions of volatiles with oxygen and combustion processes, is well visible. However, the emission of HCN and HNCO due to the lack of the *m*/*z* ion 42 disappears in the second decomposition stage. In addition, at *T*_max2_, the appearance of the *m*/*z* ion 52 may be due to the formation of cyanogen ((CN)_2_) as a result of the reactions between formed intermediate products [41,42,43]. In the case of the compounds containing the chlorine atom/atoms in their structures (**3**–**5**), the formation of HCl and/or chlorine (Cl_2_) as one of the products of the oxidation of aromatic compounds is expected. However, the formation of Cl_2_ is unlikely because the *m*/*z* ions 70, 72, and 74 are absent in the QMS spectra. Meanwhile, the presence of the *m*/*z* 36 (H^35^Cl^+^) and 38 (H^37^Cl^+^) in the QMS spectra indicates the emission of HCl as a result of the oxidation of chloroamines [44,45] in the second decomposition stage. HCl emission is also demonstrated as “jerky” bands at the range of 2640–3100 cm^−1^ in the FTIR spectra. The most expected decomposition path of the tested compounds in an air atmosphere is presented in Scheme 2.

## 3. Materials and Methods

### 3.1. A Set of the Investigated Molecules (***1***–***5***)

3-Isopropyl-8-(R-phenyl)-4-oxo-4,6,7,8-tetrahydroimidazo[2,1-*c*][1,2,4]triazines (**1**–**5**) have been used for the thermal research purposes. These molecules have been prepared from 2-hydrazono-1-(R-phenyl)imidazolidines and ethyl 3-methyl-2-oxobutanoate, as earlier described [1,4]. As previously published and patented [1,4], they have been characterized by their melting points, proton nuclear magnetic resonance spectra, attenuated total reflection Fourier transform infrared spectra, and ultraviolet spectra consistent with the assigned structures, found elemental analyses within ± 0.4% of the theoretical values, the determined retention times on HPLC columns with an endcapped octadecylsilyl as well as octadecylsilyl immobilized artificial membrane. The high purity of all compounds has been confirmed by thin-layer chromatography and high-performance liquid chromatography [1,2]. The molecular structure in the crystal of **2** has recently been published [3].

### 3.2. Differential Scanning Calorimetry (DSC)

The peak maximum melting temperatures (*T*_peak_), the onset melting temperatures (*T*_onset_), and the melting enthalpies (Δ*H*) of the tested compounds (**1**–**5**) were evaluated based on the DSC analysis using a DSC 204 apparatus, Netzsch, Selb, Germany. The sample (ca. 10 mg) was heated from room temperature to 220 °C with a heating rate of 10 K min^−1^ using an inert atmosphere (a helium gas with a flow rate of 40 mL min^−1^) and an oxidizing atmosphere (a synthetic air with a flow rate of 100 mL min^−1^). All analyses were performed in aluminum crucibles with pierced lids. 

### 3.3. Simultaneous Thermogravimetric Analysis Coupled with FTIR and QMS (TG/DTG/FTIR/QMS)

The simultaneous TG/DTG/FTIR/QMS analysis was performed with the use of a simultaneous thermal analyzer (STA 449 Jupiter F1 instrument Netzsch, Selb, Germany) coupled online with an FTIR TGA 585 analyzer (Bruker, Mannheim, Germany) and a QMS 403 C Aëolos analyzer (Netzsch, Selb, Germany). The TG apparatus was calibrated with the standard weights according to the manufacturer’s proceedings. The temperature and sensitivity calibrations with the use of the following metals (mass ca. 10 mg): In, Sn, Bi, Zn, Al, and Au were made. All the results were checked and confirmed with calcium oxalate monohydrate. Compounds **1**–**5** were heated from 40 °C to 450 °C in an inert atmosphere (helium with a flow rate of 40 mL min^−1^) and from 40 °C to 650 °C in an oxidizing atmosphere (synthetic air with a flow rate of 100 mL min^−1^). The sample (ca. 10 mg) was heated with a heating rate of 10 K min^−1^ in open Al_2_O_3_ crucibles. Simultaneously, the FTIR spectra of the emitted volatiles were collected by an FTIR TGA 585 analyzer. The FTIR spectrometer with an IR cell maintained at 200 °C was connected online to an STA instrument by a Teflon transfer line with a diameter of 2 mm and heated to 200 °C in order to avoid a condensation process of the volatiles. The FTIR spectra were gathered from 600 cm^−1^ to 4000 cm^−1^ with 16 scans per spectrum and with a resolution of 4 cm^−1^. The QMS analyzer was connected online to an STA instrument by a quartz capillary heated to 300 °C. The QMS was operated under electron ionization of 70 eV. The QMS spectra were gathered in the range of 10–150 *m*/*z*.

## 4. Conclusions

For the first time, the thermal behavior, the decomposition course, and the decomposition mechanism of a novel class of polynitrogenated heterocycles (in the solid state) in inert and oxidizing atmospheres were studied employing the DSC and simultaneous TG/DTG/FTIR/QMS analyses. In addition, the DSC measurements made it possible to check their purity.

(1) It was found that all the tested compounds melted within one narrow temperature range, and the melting point value depended on their structure. The solid–liquid phase transition of each compound was documented as one sharp peak. Their melting points were ordered as follows: the 3-Cl derivative < the 4-CH_3_ derivative < the unsubstituted compound < the 4-Cl derivative < the 3,4-Cl_2_ derivative. However, it was proven that the melting point value did not depend on the atmosphere in which the research was carried out.
(2) The TG/DTG analyses proved that the decomposition process of the studied compounds occurred in one stage in an inert atmosphere. The thermal stability of all the molecules was higher than 250 °C in inert conditions and depended on their structure. The heating of the tested compounds to 450 °C led to their complete or almost complete decomposition.
(3) The decomposition enthalpies (Δ*H_dec_*) evaluated in an inert atmosphere were dependent on the type of the substituent at the phenyl moiety. The compounds containing at least one chlorine substituent at the phenyl moiety were characterized by lower Δ*H_dec_* values than the remaining molecules without chlorine atoms in their structure. This proved that the presence of the electron-withdrawing substituent/substituents in the structure reduces the heat energy needed for the decomposition of the tested compounds.
(4) On the basis of the FTIR and QMS analyses, it was found that the main decomposition products of the tested compounds were NH_3_, HCN, HNCO and its derivatives, CO_2_, CO, H_2_O, aromatic amine derivatives, alkenes (for compounds **1**–**5**), and HCl (for the compound **5**). The type of volatiles indicated the random breaking of the C–N and the C–C bonds in the structures of the studied compounds and the formation of intermediates.
(5) In turn, the conducted research confirmed that the decomposition process of the tested molecules occurred in at least two stages in an atmosphere of synthetic air. The thermal stability was higher than 260 °C in an oxidizing atmosphere and, as a rule, higher than that evaluated in an inert atmosphere. Hence, it is probable that oxygen may delay the decomposition process of these polynitrogenated heterocycles by enhancing the decomposition activation energy of newly formed radical species. Their thermal stability in the presence of a synthetic air atmosphere was ordered as follows: the 4-CH_3_ derivative (264 °C) < the unsubstituted compound (275 °C) < the 4-Cl derivative (286 °C) < the 3-Cl derivative (290 °C) < the 3,4-Cl_2_ derivative (300 °C), taking into account the values of their initial decomposition. All the tested compounds completely decomposed after their heating to 650 °C.
(6) The decomposition enthalpies in the first decomposition stage (Δ*H_dec_*_1_) and in the second decomposition stage (Δ*H_dec_*_2_) evaluated in a synthetic air atmosphere showed significantly different values. The lower Δ*H_dec_*_1_ values, as compared to Δ*H_dec_*_2_ values, indicated a different nature of the decomposition process in both stages. Mainly, the chemical reactions between the intermediate decomposition products and/or oxygen were expected in the first decomposition stage. In turn, during the second decomposition stage, the combustion processes were the main processes.
(7) The emission of the following gases: NH_3_, CO_2_, CO, HCN, HNCO, H_2_O, N_2_, NO_2_, NH_2_OH, and some aromatics in the first decomposition stage were observed. While during the second decomposition stage, the emission of CO_2_, CO, H_2_O, HCN, N_2_, NO_2_, NH_2_OH, (CN)_2_ (for compounds **1**–**5**) and HCl (for compounds **3**–**5**) was confirmed. The emission of the additional volatiles such as N_2_, NO_2_, NH_2_OH, and (CN)_2_ indicated that the decomposition process of these compounds was more complicated in an oxidizing atmosphere. It included the simultaneous random breaking of the C–N and the C–C bonds combined with formed volatiles-oxygen reactions and combustion processes of the volatiles and residues.
(8) It was proven that the investigated compounds are materials with high thermal stability and purity, which makes them attractive for pharmaceutical applications. The research results are important from a practical point of view and, therefore, will be helpful in selecting the most promising compounds for further clinical trials and in evaluating the conditions of their storage and processing, as well as their influence on the environment under the heating. From a scientific point of view, the information on the decomposition pathways of thermally stable heterocyclic drug-like molecules, especially those of small molecular mass, is an important contribution to the current state of knowledge.

## Figures and Tables

**Table 1:** The DSC data for compounds **1**–**5** (helium and synthetic air atmospheres).

Sample	R	Melting Process					
		Helium			Synthetic Air		
		*T*_onset_/°C	*T*_peak_/°C	Δ*H*/J/g	*T*_onset_/°C	*T*_peak_/°C	Δ*H*/J/g
**1**	H	198.9	202.8	81.8	197.2	201.8	154.6
**2**	4-CH_3_	194.8	197.7	74.7	193.1	197.1	151.3
**3**	3-Cl	148.7	152.7	46.5	148.7	152.9	122.3
**4**	4-Cl	200.0	203.5	57.6	198.3	202.4	125.5
**5**	3,4-Cl_2_	200.8	204.3	61.2	199.8	203.8	128.9

R—the substituent at the phenyl ring.

**Table 2:** The TG/DTG data for compounds **1**–**5** (a helium atmosphere).

Sample	R	Degradation Process				
		First Decomposition Stage				
		*T*_5%_/°C	*T*_max1_/°C	Δ*m*_1_/%	Δ*H_dec_*/J/g	rm/%
**1**	H	253	326	98.6	275.9	1.4
**2**	4-CH_3_	266	340	99.0	263.8	1.0
**3**	3-Cl	259	337	100.0	234.4	0.0
**4**	4-Cl	266	340	97.5	239.7	2.5
**5**	3,4-Cl_2_	269	350	100.0	208.4	0.0

R—the substituent at the phenyl ring, rm—residual mass at 450 °C.

**Table 3:** The TG/DTG data for compounds **1**–**5** (a synthetic air atmosphere).

Sample	R	Degradation Process						
		First Decomposition Stage				Second Decomposition Stage		
		*T*_5%_/°C	*T*_max1_/°C	Δ*m*_1_/%	Δ*H_dec_*_1_/J/g	*T*_max2_/°C	Δ*m*_2_/%	Δ*H_dec_*_2_/J/g
**1**	H	275	343	87.6	376.2	589	12.4	2010
**2**	4-CH_3_	264	349	90.2	341.6	584	9.8	3053
**3**	3-Cl	290	356	78.9	462.8	562	21.1	3267
**4**	4-Cl	286	355	76.5	445.6	576	23.5	3095
**5**	3,4-Cl_2_	300	358	64.9	438.3	555	35.1	3280

R—the substituent at the phenyl ring, rm—residual mass at 650 °C.

## Data Availability

The data presented in this study are available on request from the authors.

## Sample Availability

The samples of the investigated compounds are available from the authors.
